# Shallow groundwater near a deep-well CO_2_ storage site—15 years of stable water quality for agricultural use

**DOI:** 10.1007/s11356-025-35906-6

**Published:** 2025-01-21

**Authors:** Zibo Zhou, Kexin Zhang, Damien L. Callahan, Wendy Timms

**Affiliations:** 1 School of Engineering, Deakin University, Waurn Ponds, Geelong, VIC 3216 Australia; 2 School of Life and Environmental Sciences, Deakin University, Burwood, Melbourne, VIC 3125 Australia

**Keywords:** Long-term groundwater quality variations, Groundwater quality assessment, CO_2_ injection site, Hydrogeochemical process, Water for agricultural usage

## Abstract

Injecting CO_2_ into deep geological formations can be an effective carbon removal and storage technology to mitigate global climate change. Interaction of injected CO_2_ with rock formations changes pH and hydrochemistry within the deep injection zone (> 800 m depth). However, cap rocks and multiple tight aquitards typically act as barriers to protect the shallow aquifer from changes in the injection zone. Monitoring and evaluation of shallow groundwater quality are essential to verify that carbon capture storage projects (CCS) do not impact the near-surface environment. This study investigated shallow groundwater quality using long-term data (2006–2023) from a regular monitoring program at the Otway International Test Centre (OITC) in Victoria, Australia. It was found that shallow groundwater quality was stable over at least 15 years, during which time three phases of CO_2_ injection into a deep storage zone occurred. The results highlighted groundwater quality complied with guidelines of Food and Agriculture Organization (FAO) and Australian water quality guidelines. Minor and localised changes observed in salinity or pH in shallow monitoring piezometers were caused by natural processes. Moreover, a wide range of groundwater quality indicators were evaluated. The results demonstrated that the groundwater quality of shallow aquifers (< 80 m) at OITC is suitable for agriculture. The study provides assurance and confidence to stakeholders that the quality of the near-surface environment has not been impacted by CO_2_ injection into confined formations and no pollution has been detected. Although numerous CCS sites around the world are subject to monitoring, no evidence of changes in shallow groundwater quality has been reported that could be traced to CO_2_ injection in confined formations at > 800 m depth.

## Introduction

Carbon capture and storage (CCS) plays a key role in global climate change mitigation, which involves the capture of CO_2_ emission and subsequent injection into geological formation for permanent storage (Solomon et al. 2008; Zheng et al. 2021; Global CCS Institute 2022). A recent review paper highlighted several critical directions for future CCS research including monitoring and early warning of CCS storages, and improving public awareness (Ren et al. 2023). CCS projects have been operated and monitored over two decades at numerous sites around the world (Zheng et al. 2021; Global CCS Institute 2022). As of September 2022, the total capacity of CCS projects in development was 244 million tonnes per annum (Mtpa) of carbon dioxide (CO_2_) (Global CCS Institute 2022).

During the evaluation phase of CCS reservoirs, the petrophysics of fluids and seals or caprocks are investigated. The evaluation and storage optimisation of reservoirs for CO_2_ containment is important to prevent leakage of CO_2_, which could impact the groundwater quality in the injection zone (Kharaka et al. 2010; Lions et al. 2014; Qafoku et al. 2017; Zheng et al. 2021; Xiao et al. 2023). Possible risk of CO_2_ injection influencing on the local near-surface environment over decadal scale has been reviewed by several studies. The major risks are related to the possibility of CO_2_ leakage via geological faults (e.g. Feitz et al. (2022); Choi et al. (2023)) and relatively acidic environment to aquifers (e.g. mobilisation of metals) that may be caused by potential CO_2_ leakage (e.g. Qafoku et al. (2017); Zheng et al. (2021); Xiao et al. (2023)).

Groundwater is an important resource of the ecosystem and plays a vital role in supporting human activities, agriculture, and industrial development, and maintaining the health of the riverine ecosystem. It is particularly important in dry and semi-arid areas (e.g. Scanlon et al. (2023)). Good groundwater quality is essential for a variety of purposes such as human usage and agricultural activities (e.g. Scanlon et al. (2023)). Hence, determining the quality of groundwater in different environmental conditions, whether or not related to CCS projects, will have critical implications for urbanisation, human population growth, and agricultural activities (Zheng et al. 2021; Scanlon et al. 2023).

### Potential effects of CO_2_ on groundwater quality

The effects of CO_2_ on groundwater quality occur due to reactions of CO_2_ with the aquifer matrix and have been observed or quantified by lab-based tests, field-based tests, and modelling (e.g. Kharaka et al. (2010); Lions et al. (2014); Zheng et al. (2021); Li et al. (2022); Xiao et al. (2023)). Dissolved CO_2_ and formation of carbonic acid decrease pH of groundwater, driving the hydrogeochemical reactions with the aquifer matrix. Groundwater with pH < 7 could dissolve carbonate, mobilise major and minor elements (e.g. Ca, Mg, Na, Ba, Fe, Al), and drive cation exchange processes (e.g. Kharaka et al. (2010)). Trace metals can be mobilised due to desorption, ion exchange reactions, and the dissolution of a variety of sediments and rock including carbonates, iron oxides, oxyhydroxides, and sulphides (Lions et al. 2014; Delkhahi et al. 2020; Zheng et al. 2021; Pearce et al. 2022). Several laboratory-based and modelling studies evaluated the potential for trace metal mobilisation in acidified groundwater. For example, Zheng et al. (2021) mentioned that elevated concentrations of Pb, As, Cd, and U under acidic conditions could be a water quality issue. The study by Qafoku et al. (2017) also suggested that mobilisation of As, Pb, Ba, Se, and Cd may degrade groundwater quality. A water-saturated column experiment that was conducted by Bacon et al. (2016) simulated the trace metals release and mobilisation when interacting with the CO_2_-rich solution. In that study, the concentrations of trace metals (As, Ba, Cr, Cu, Pb Sb, Se) rapidly increased following the first interaction with CO_2_ due to pH-driven chemical reactions. The concentrations of trace metals gradually decreased with time due to the effect of kinetic desorption and most of the metal concentrations were below the local maximum concentration limits (Bacon et al. 2016). Field test studies from the US at CCS demonstration sites in Montana (the Zero Emission Research and Technology (ZERT) field site) and Mississippi observed the trace metal release process; however, concentrations did not exceed the local environment’s maximum concentration limits (Kharaka et al. 2010; Yang et al. 2015). There is no reported evidence for deep-well injection of CO_2_ degrading the quality of water resources near or at the ground surface case for degraded water quality despite monitoring and assessments. However, it is essential to continue monitoring, to evaluate the potential mobilisation of pollutants and dynamic conditions that may trigger water quality limits, ensuring that any temporary influences do not accumulate over the long term (e.g. Ghosh and Jha (2024)). Additionally, the potential for leakage of CO_2_ into aquifers is site-specific and therefore pre-assessments and monitoring are needed at each site (e.g. Qafoku et al. (2017); Zheng et al. (2021)).

As reported in previous studies, CO_2_ injection into aquifers could cause changes in alkalinity, electrical conductivity (EC), and total dissolved solids (TDS) of groundwater (e.g. Peter et al. (2012); Lions et al. (2014); Qafoku et al. (2017)). Due to calcite dissolution and pH-driven adsorption or desorption reactions, these parameters may present a pulse-like trend, with rapid response to CO_2_ exposure followed by a gradual return to the natural background (Peter et al. 2012; Qafoku et al. 2017; Zheng et al. 2021). By injecting CO_2_, naturally occurring brine may also be displaced upward into an adjacent groundwater aquifer if there is a gradient upwards that is greater than the downwards gradient associated with a denser brine containing dissolved CO_2_ (Qafoku et al. 2017; Zheng et al. 2021). The brine’s salinity and the presence of contaminants (i.e. As) might degrade groundwater quality if it migrates into lower salinity aquifers (Zheng et al. 2021).

While the potential impacts of CO_2_ on the quality of overlying water aquifers have been assessed over the last decade, the CO_2_ that caused concerns may not be related to the CCS operation (Lions et al. 2014; Zheng et al. 2021; Xiao et al. 2023). For example, an allegation reported that injected CO_2_ was leaking into the soil and groundwater in Saskatchewan, Canada, in 2011; however, subsequent investigation and advanced data collection found no evidence of leakage at this CCS site and instead identified the CO_2_ to be the result of natural processes (Gilfillan et al. 2017). The study collected water from four groundwater bores and surrounding the alleged leakage location. A series of δ^13^C_DIC_ and noble gas parameters (e.g. ^3^He/^4^He, ^20^Ne, ^36^Ar, ^40^Ar, and Kr) were analysed, with the study concluding that elevated CO_2_ concentrations were from biological origin (Gilfillan et al. 2017). It is also emphasised that a good understanding of baseline concentrations and sources of natural CO_2_ is therefore important.

Most CCS-related water quality studies either evaluated relatively short period of data collection (i.e. less than 5 years) or focused on characterising mechanisms of geochemical interaction associated with CO_2_ injection (Lions et al. 2014; Yang et al. 2015; Qafoku et al. 2017; Gholami et al. 2021; Li et al. 2022; Xiao et al. 2023). For example, a study by Yang et al. (2015) collected groundwater chemistry data (i.e. major ions and trace elements) from 2008 to 2014 at a CCS site in Mississippi, US, reporting that no geochemical parameters exceeded the natural background variability. Gholami et al. (2021) summarised the effect of CO_2_ injection with different reservoir rocks and concluded that combinations of geochemical reactions, pressure, and temperature can induce potential CO_2_ leakage along weakness in caprocks or aquitards either through wells or along geological faults. Although there have been decades of operational experience at several CCS projects around the world, studies demonstrating long-term monitoring data for shallow groundwater quality remain scarce. This study initially evaluated long-term shallow groundwater quality variation using over 15 years of data at a CCS site.

### Study objectives

This study uses more than 15 years of regular monitoring data since 2006 at a CCS site, to provide long-term trends of hydrochemistry in shallow groundwater and groundwater quality for local agricultural usage (e.g. irrigation and livestock). Groundwater sampling is part of the broader program for monitoring and assurance of the Otway International Test Centre (OITC) in the Otway Basin, southwestern Victoria, where multiple hydrochemical indicators and tracers were used during deep-well CO_2_ injection (Boreham et al. 2011; de Caritat et al. 2013).

The objective of this study is to highlight how groundwater quality can be monitored for beneficial use by agriculture overlying and near a deep-well CO_2_ injection site. It was beyond the scope of this paper to evaluate the unlikely possibility of migration of CO_2_ upwards from the target reservoir using tracers such as by de Caritat et al. (2013). In contrast, this paper evaluates long-term groundwater quality for agriculture and any changes observed in salinity, pH, trace metals, and water quality indicators for irrigation use. The geochemical processes contributing to water quality, and the potential for dissolution and mobilisation of trace elements are considered. This study, for the first time, provides long-term data evaluation on near-surface water resources at a demonstration CCS site, that can be useful to support future large-scale CCS projects.

## Setting

The Otway Basin is in southeastern Australia with an area of approximately 155,000 km^2^, 80% of which is located offshore and extends eastward into Port Philip Bay and westward into South Australia (Iverach et al. 2020). The basin comprises a series of sedimentary and volcanic rocks from the Mesozoic and Cenozoic eras that are 10 km thick and include several important aquifers (Clarke et al. 2015). A primary structural restraint on the basin was created by Australia’s continental rifting from Antarctica, which started in the late Jurassic (Bush 2009). During the Pliocene, the region was largely covered by alluvial outwash deposits of the Hanson Plain Sand, and aerial exposure caused the Timboon Surface, which has karstic topography on Port Campbell Limestone. Large basalt outpourings blanketed the basin’s northern edge, and several scoria cones and maar craters were created by younger, more explosive volcanism (Bush 2009; Clarke et al. 2015). Agriculture is the main regional land use (81%), mostly for broadacre cropping, with dairying and grazing coming in second and third. Native woodland (16%), pine forest (2%), and urban and industrial development (< 1%) make up the remaining land use (Iverach et al. 2020). The region has a temperate climate with winter precipitation being heavier and increasing towards the shore. The Southern Ocean’s vicinity regulates both the maximum and lowest temperatures of both seasons in the region, which has hot, dry summers and cold, wet winters. The region’s yearly average temperature falls between 17 and 20 °C (Bush 2009).

**Fig. 1:**
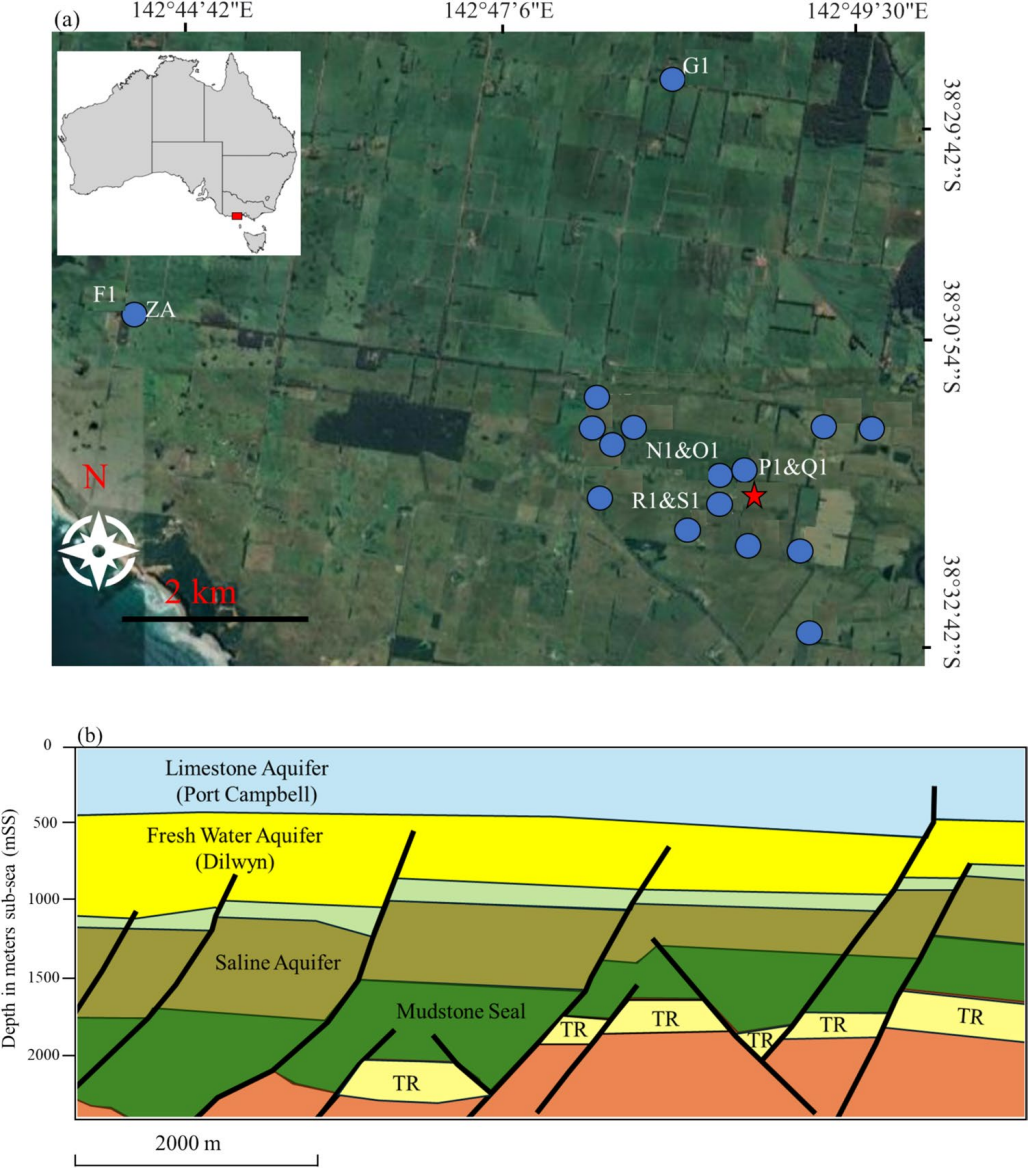
**a** Google Earth image (©Google LLC) of groundwater sampling sites at OITC in Victoria, southeast Australia. OITC is the CO2CRC Otway International Test Centre and is indicated by the star symbol. Government bores (F1, G1, and ZA) and CO2CRC piezometers (N1, O1, P1, Q1, R1, and S1) are presented with letters. Landholder bores are shown with blue dots. **b** Schematic cross-section of geology and fault at the OITC. The cross-section is modified based on Boreham et al. (2011). Limestone aquifer does not differentiate the shallow formations: Hesse Clay, Port Campbell Limestone, and Gellibrand Marl. TR = target reservoir

The CO2CRC Otway International Test Centre (OITC) is located in the onshore Otway Basin in southwestern Victoria, Australia, on a private farm (Fig. 1a). Near-surface geologic strata at the CO2CRC OITC are comprised of the Hesse Clay, the Port Campbell Limestone, the Gellibrand Marl, and dune deposits (de Caritat et al. 2013; Fig. 1b). This site was developed to demonstrate CO_2_ injection technologies and allows industry and academia to investigate and overcome engineering challenges and assess technical performance under operational conditions, prior to embarking on a commercial CCS project. To April 2021, OITC has successfully stored a total of 96,000 tonnes CO_2_ into depleted gas reservoir (at 2000 m depth) and saline aquifer (at 1500 m depth). Stage 1 Project was from April 2008 to September 2009, Stage 2 Project was from May 2011 to May 2019 (separated into Stage 2B, 2B Extension, and 2C), and Stage 3 Project was from July 2019 to April 2021 (Nourollah et al. 2023).

Regional groundwater flow direction within the Port Campbell Limestone and Dilwyn Formation aquifers has been described as south-westerly and towards the coast (de Caritat et al. 2013; Fig. 1b). On the other hand, the groundwater within the limestone aquifer in the area immediately adjacent to the OITC flows towards the northeast and in the direction of the nearby Curdies River (de Caritat et al. 2013). General regional hydraulic gradients ranged from 100 m inland to 20 m close to the coast with minor impacts from seasonal rainfalls (de Caritat et al. 2013). Groundwater extracted from the unconfined to semiconfined Port Campbell Limestone serves various purposes such as irrigation, dairy farming, and domestic use. CO_2_-rich fluid was injected into the target reservoir more than 2000 m depth below a thick mudstone seal. Groundwater bores are in the shallow formations including Hesse Clay, Port Campbell Limestone, and Gellibrand Marl (Fig. 1b). Groundwater bores are above the deeper and confined aquifer known as the Dilwyn Aquifer, which is situated well above the injection zone separated by alternating layers of aquitards and aquifers as shown in Fig. 1b (Boreham et al. 2011; de Caritat et al. 2013).

## Methods

### Bore selection and sampling

Groundwater samples were collected annually from 18 bores and piezometers near the CO2CRC Otway International Test Centre (OITC) in southwestern Victoria since 2006 (Fig. 1a). A number of groundwater bores were selected at OITC as reference bores to consider the long-term trends. These bores were chosen based on the localities and purposes, which were grouped into Victorian government monitoring bores (G1, F1, and ZA), CO2CRC monitoring piezometers (N1, O1, P1, and R1), and selected reference bores that were located in down-gradient of an injection well (de Caritat et al. 2013). These reference bores also include some landholder bores that are used for agriculture. Victorian government bores are located several kilometers from the potential extent of the deep CO_2_ storage zone, suitable for determining natural background variability. The CO2CRC monitoring piezometers near the injection wells provide information on near-surface geology/hydrogeology (Radke et al. 2022).

For QA/QC purposes following standard practices, several volumes of water were purged from the bore prior to sampling using a submersible Grundfos MP1 pump that was lowered into the bore casings or using a disposable bailer.

### Analytical techniques

Water quality parameters were measured using a hand-held (Orion Star A329) portable water quality meter in the field. The parameters include electrical conductivity (EC), dissolved oxygen (DO), pH, oxidation–reduction potential (ORP), and temperature. The water quality meter was calibrated each morning prior to sampling.

A Hach© DR1900 spectrophotometer was used to measure total alkalinity (expressed as CaCO_3_), total iron, ferrous iron (Fe^2+^), sulphate (SO_4_^2−^), and sulphide (S^2−^) concentrations at the time of sampling. For precision of total alkalinity, the colorimetric (Method 10239, TNTplusTM870) method is also applied with lower and upper detection limits of 25 mg/L and 400 mg/L, respectively. Total iron was measured using the phenanthroline (Method 10229, TNTplusTM858) method with lower and upper detection limits of 0.2 mg/L and 6.0 mg/L, respectively. Ferrous iron was measured using the 1,10 phenanthroline (Method 8146, powder pillows) method with lower and upper detection limits of 0.02 mg/L and 3.00 mg/L, respectively. Sulphate was measured using the turbidimetric (Method 10227, TNTplus®864) method with lower and upper detection limits of 40 mg/L and 150 mg/L, respectively. Sulphide was measured using the USEPA Methylene Blue (Method 8131, Reagent Solution) method with lower and upper detection limits of 5 µg/L and 800 µg/L, respectively. Accuracy checks of the spectrophotometer were performed using standards prepared each morning prior to sampling. At a number of locations, the measured value of total alkalinity exceeded the upper range limit (400 mg/L) of the spectrophotometer. Concentrations of this parameter were thus subsequently re-analysed using a Hach© digital titrator alongside a pH meter. The complete dataset will be available on request.

Cation and anion concentrations were determined at Monash University using an inductively coupled plasma optical emission spectrometry (ICP-OES; Thermo Fischer) and an ion chromatograph (Thermo Fischer), respectively. Samples were filtered through 0.45-µm cellulose nitrate filters and acidified to pH < 2 for cation measurements and filtered and unacidified for anion measurements. Overall precision (σ) of major ion concentrations is 2–5% based on replicate analyses. The charge balance error (CBE) was computed to check the quality of the chemical analysis, and 17 samples with > 10% error were not included in further evaluation.

Samples collected for trace elements were acidified at the time of sampling using concentrated nitric acid (HNO_3_). An ICP-mass spectrometer (ICP-MS; Perkin Elmer Nexion350) was used to analyse trace elements at Deakin University. The number of trace elements with analytical standards increased to approximately 58 elements as part of the 2023 groundwater survey, which were generally at concentrations near or below the detection limit and will be discussed in this paper.

For QA/QC purposes, duplicate analyses were performed on approximately 10% of all primary samples and NIST (National Institute of Standards and Technology) standards were used for validation. Sample procedures also include pre-sampling purging for stable field parameters, usage of new filters and triple rinsing sample containers, and use of high-quality chemicals and distilled deionised water.

### Groundwater quality indicators for agriculture

In this study, several water quality indicators for agricultural sustainability were adopted to evaluate the groundwater quality status in addition to basic measurements such as salinity and pH. Those include sodium adsorption ratio (SAR), soluble sodium percentage (SSP), magnesium hazard (MH), permeability index (PI), and Kelley’s ratio (KR) of major cations. These indicators were widely used in water quality studies to evaluate fit-for-purposes of irrigation usage (e.g. Mohammed et al. (2022)) and can be calculated as follows where all ions are in milliequivalent per Liter (meq/L):

1 $${\text{SAR}}=\frac{{\text{Na}}^{+}}{\sqrt{\frac{{\text{Ca}}^{{2}+}{\text{Mg}}^{{2}+}}{2}}}$$

2 $${\text{SSP}}=\frac{{\text{Na}}^{+}\text{+}{\text{K}}^{+}}{{\text{Ca}}^{2+}\text{+}{\text{Mg}}^{2+}\text{+}{\text{Na}}^{+}\text{+}{\text{K}}^{+}}\times {100}$$

3 $${\text{MH}}=\frac{{\text{Mg}}^{+}}{{\text{Ca}}^{2+}\text{+}{\text{Mg}}^{2+}}\times {100}$$

4 $${\text{PI}}=\frac{{\text{Na}}^{+}\text{+}\sqrt{{\text{HCO}}_{3}^{-}}}{{\text{Ca}}^{2+}\text{+}{\text{Mg}}^{2+}\text{+}{\text{Na}}^{+}}\times {100}$$

5 $${\text{KR}}=\frac{{\text{Na}}^{+}}{{\text{Ca}}^{2+}\text{+}{\text{Mg}}^{2+}}\times {100}$$

## Results and discussion

### Long-term variations in groundwater quality

**Fig. 2:**
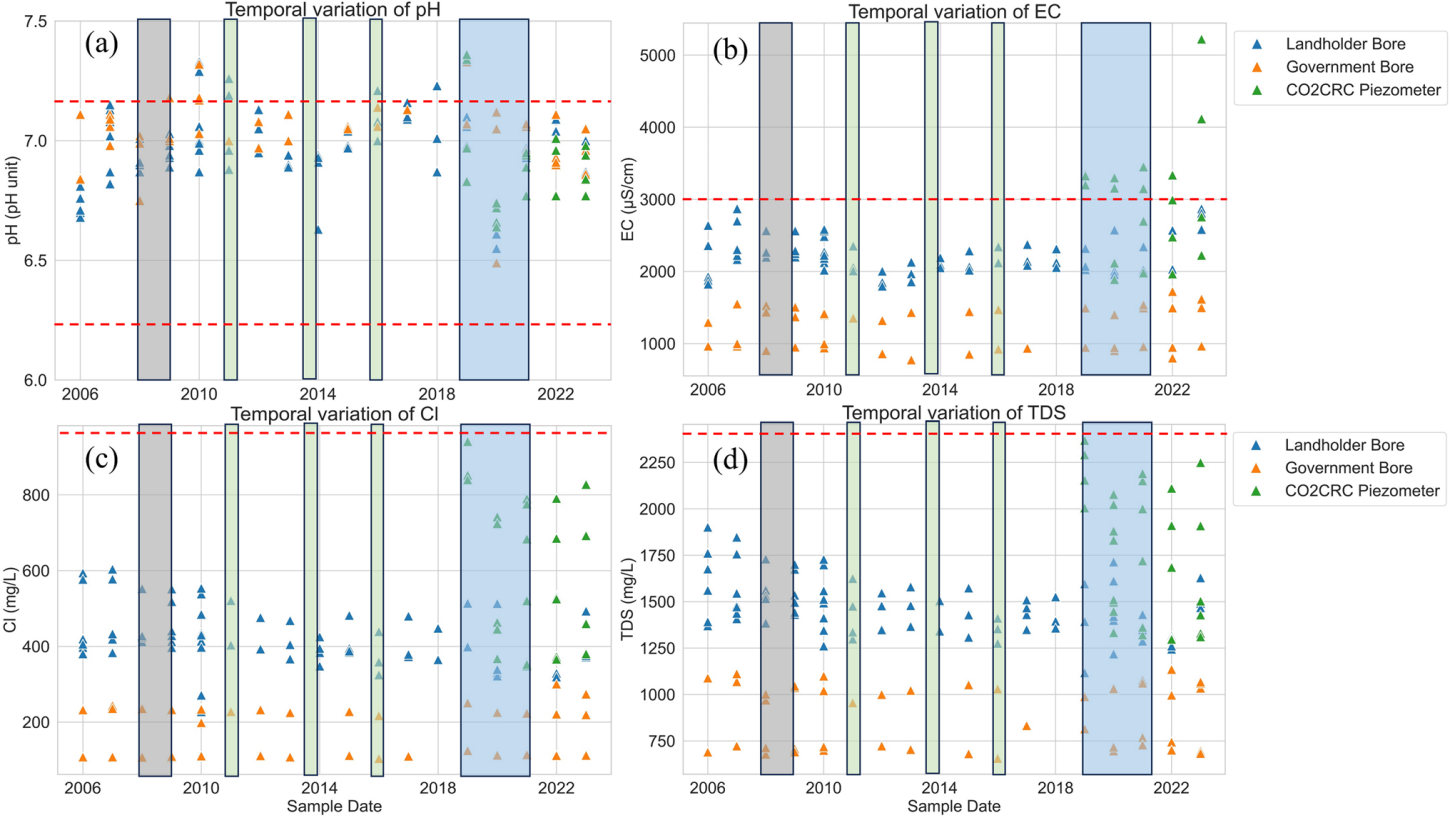
pH, EC, Cl, and TDS values from selected groundwater bores/shallow piezometers at OITC (2006–2023). Shading areas represent the CO_2_ injection projects (grey = Stage 1 injection; green = Stage 2 injection; blue = Stage 3 injection). Red dash lines present the natural background variabilities of limestone aquifers that were summarised by Clarke et al. (2015) and Iverach et al. (2020)

Time-series variations of pH, EC, Cl, and TDS concentrations of groundwater are shown in Fig. 2. Between 2006 and 2023, pH was stable within the range of 6.4 to 7.3 although there was a short-term decline between the 2019 and 2020 sampling. The largest pH change (7.4 to 6.6 and 7.3 to 6.5 respectively) occurred at shallow piezometer sites O1 and R1 and the government bore F1 (Figs. 1a and 2a). The pH change in landholder bores was from 7.1 to 6.6 (Fig. 2a). By the next annual sampling campaign, the pH returned to the natural background values (pH 6.2 to 7.2) of this area (Bush 2009). The temporary reduction in pH in shallow monitoring bores during this period, particularly, was likely related to recharging water from rainfall (which tends to be slightly acidic), since antecedent conditions prior to sampling indicate relatively wet conditions for the 2020 sampling campaign. There was a total rainfall of 178 mm in the 60 days prior to sampling in June 2020, in contrast to total rainfall of 63 mm in 60 days before the 2019 sampling campaign (which was also a drier year than 2020, Bureau of Meteorology 2024). The influence of rainfall recharge was indicated by variations of EC and stable isotopes (^18^O and ^2^H) data in 2019 and 2020 at those sites (supplementary material). EC values at the government bore F1 decreased from 1500 to 1402 mS/cm and at shallow piezometers (O1 and R1) EC declined from 3456 to 3157 mS/cm. This is consistent with stable isotope data that the values in 2020 were depleted and closer to average rainfall values. This evidence demonstrated that the changes in pH are more likely to be caused by rainfall impacts than CO_2_ injection activities.

EC values at government monitoring bores and landholder bores were relatively stable from 2006 to 2023 (Fig. 2b) and remained below 3000 mS/cm. However, higher EC was observed in newly constructed shallow monitoring piezometers (i.e. bores O1, P1, and R1 with average values of 3367 mS/cm, 2438 mS/cm, and 3587 mS/cm respectively). Salinity increased at O1 and R1 to 4118 and 5223 µS/cm respectively in 2023 (Fig. 2b). Similarly, the trend of Cl concentrations and TDS were different in CO2CRC shallow monitoring piezometers compared to government bores and landholder bores (Fig. 2c and d). While Cl and TDS concentrations in CO2CRC shallow monitoring piezometers were higher (shown in green triangles) since they were installed in 2019, these were still below the natural background variabilities (i.e. Cl 400–1700 mg/L and TDS 650–2400 mg/L respectively; Fig. 2c and d). The recent change in EC could be related to the construction of these shallow monitoring bores or specific geochemical processes. Details of a geochemical evaluation are discussed in the ‘Recent geochemical trends at shallow monitoring piezometers’ section. Overall, the data from 2006 to 2023 indicated that long-term general groundwater quality from selected sites remained stable and was within the natural background variability. This demonstrates that CO_2_ injection activities have no negative impacts on shallow groundwater pH and salinity at OITC.

### Groundwater quality suitability for agriculture

#### Australian standard for livestock and irrigation

Shallow groundwater pH, EC, and major ion chemistry were summarised and compared with widely used standards indicating that the groundwater from 2006 to 2023 was generally suitable for agricultural uses (Table 1). It was also observed that groundwater temperature ranged from 12.6 to 33.2 and groundwater was generally oxygenated (0.02 to 13.02 mg/L) and had no odour or colour. FAO (Food and Agriculture Organization) irrigation water guidelines (Ayers and Westcots 1994) and Australian standards for livestock and irrigation purposes (ANZECC and ARMCANZ 2000) have been added for comparison (Table 1).

**Table 1:** Summary of statistics of groundwater water quality parameters (2006–2023)

Parameter	Min	Max	Average	SD (σ)	FAO STD for irrigation	AU STD for irrigation ^1^	AU STD for livestock
EC (µS/cm)	498	5223	1799	668	0–3000	1300–2900	< 5970
pH	6	9.4	7.1	0.4	6.5–8.4	6–8.5	6.5–8.5
HCO_3_ (mg/L)	203	655	446	90	0–610	n/a	n/a
Cl (mg/L)	47	964	335	176	0–1050	< 350	< 4000 for beef cattle 1600 for dairy cattle
Na (mg/L)	53	556	205	97	0–920	230–460	n/a
Ca (mg/L)	4.2	260	142	45.3	0–400	n/a	Up to 1000
Mg (mg/L)	5.7	53.4	25.3	9.4	0–60	n/a	n/a
NO3 (mg/L)	0.01	75.61	6.65	11.37	0–30	< 125 ^2^	< 400
SO4 (mg/L)	0.4	208	54.3	31.8	0–960	n/a	< 1000
K (mg/L)	0.16	25.1	4.70	4.51	0–2	n/a	n/a
TDS (mg/L)	403	2417	1230	382	0–2000	870–1950	< 4000 for all and 2500 for dairy cattle

^1^The values from Australian standard were moderately tolerant range

^2^The value includes all inorganic forms of nitrogen present in water

Overall, the mean values of groundwater parameters at OITC complied with the range of FAO standard except for potassium (K) that the average concentration exceeds the FAO standard. Mean potassium concentrations that were higher than the suggested value of FAO for irrigation water could influence soil permeability (i.e. clay swelling and dispersion) (Smith et al. 2015). However, this is not a concern as the high concentrations are from government monitoring bores, which is not used for agricultural purposes. In addition, the average values in all parameters are in the range of Australian water quality standard guidelines for livestock and/or irrigation activities while some of the maximum values were over the range. For example, the highest value of EC (5223 mS/cm) was measured at a shallow piezometer that exceeded both FAO and Australian standards for irrigation purposes. However, this is not a concern as the high concentrations are from a piezometer, which is not used for agricultural purposes The relatively high EC here is discussed further in the ‘Recent geochemical trends at shallow monitoring piezometers’ section.

#### Water quality indicators for agriculture

**Fig. 3:**
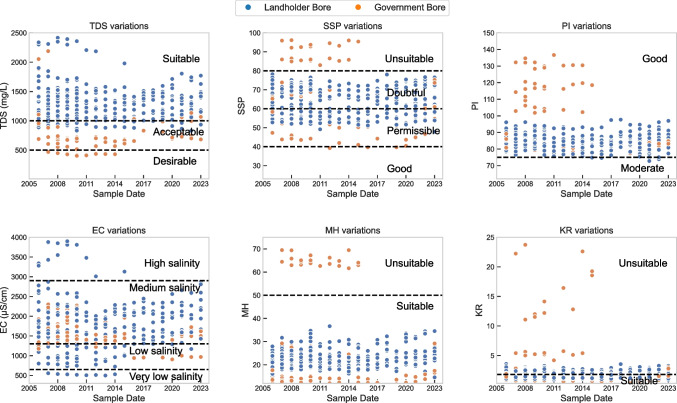
Classifications of key groundwater quality indices (2006–2023)

The relatively stable groundwater quality over time also proved beneficial for irrigation. Key water quality indices for irrigation were evaluated between 2006 and 2023 for landholder and government bores, but not for CO2CRC’s shallow monitoring piezometers. These indices were sodium adsorption ratio (SAR), soluble sodium percentage (SSP), magnesium hazard (MH), permeability index (PI), and Kelley’s ratio (KR) together with TDS and EC (Fig. 3). Different indicative lines were showed in Fig. 3 for evaluation of the key groundwater quality indices. Statistical parameters are also summarised in Table 2 to indicate the distribution of water quality hazard classifications.

**Table 2:** Summary of groundwater quality assessment parameters in the study area (landholder and government bores). The group that accounted for the largest proportion was italic and highlighted

Parameters	Class	Hazard	No. of samples	Percentage (%)
TDS	< 500	Desirable	11	2.6
	500–1000	Acceptable	89	21.0
	***1000–3500***	***Suitable***	***324***	***76.4***
	3500–13000	High salinity	0	0.0
EC	< 650	Very low salinity	11	2.6
	650–1300	Low salinity	72	16.7
	***1300–2900***	***Medium salinity***	***329***	***77.6***
	2900–5200	High salinity	13	3.1
	5200–8100	Very high salinity	0	0
SAR	** < *10***	***Low sodium***	***404***	***95.3***
	10–18	Medium sodium	17	4.0
	18–26	High sodium	3	0.7
	> 26	Very high sodium	0	0.0
SSP	< 20	Excellent	0	0.0
	20–40	Good	3	0.7
	40–60	Permissible	157	37.0
	***60–80***	***Doubtful***	***241***	***56.8***
	> 80	Unsuitable	23	5.4
MH	** < *50***	***Suitable***	***401***	***94.6***
	> 50	Unsuitable	23	5.4
PI	** > *75***	***Good***	***419***	***98.8***
	25–75	Moderate	5	1.2
	< 25	Unsuitable	0	0.0
KR	< 1	Suitable	28	6.6
	** > *1***	***Unsuitable***	***396***	***93.4***

Salinity hazards, which are commonly indicated by TDS concentrations or EC values, could prevent the crop from absorbing enough water. Consequently, a substantial reduction in water intake causes the plant to slow down its pace of growth (e.g. Mohammed et al. (2022)). TDS values of groundwater ranged from 403 to 2417 mg/L (1194 ± 356 mg/L) and were in the range of desirable to suitable classifications based on the US Salinity standards (1954). The EC values have a similar scenario in that most of the samples are classified between low to medium salinity, which indicates no significant salinity issues.

SSP is also referred to as sodium percentage (Na %) and it presents the percentage of Na out of the total cations. If Na-rich water is frequently used for irrigation, the soil could be plastic and sticky when gets wet, and clods and crusts will be formed in dry conditions (Mohammed et al. 2022). SSP is commonly classified into five categories (Table 2). SSP from groundwater samples at OITC site ranged from 39 to 96 with a mean value of 64 ± 11. According to the classifications, 37.0 to 56.8% of water samples fell within the permissible to doubtful category, and 5.4% of the samples were considered unsuitable for irrigation purposes as shown in Table 2. Similarly, these high values were also from 2007 to 2015 datasets and mainly from government monitoring bores. MH ratios represent the proportion of magnesium, and the ratio exceeding 50 can be seen as harmful and unsustainable as soil will become more alkaline and will have impacts on crop yields (e.g. Mohammed et al. (2022)). MH of groundwater samples was from 11 to 69 (24 ± 11) and 94.6% of water samples were suitable (Table 2). The PI can indicate the rate at which water deteriorates soil structure; a high ratio denotes a high rate of deterioration. Poor soil permeability prevents water from flowing downward, which leads to poor drainage. It also frequently creates a surface crust that inhibits seed emergence and germination (Singh et al. 2020). The percentage of groundwater samples within the ‘good’ classification in PI is 98.8% (Table 2).

**Fig. 4:**
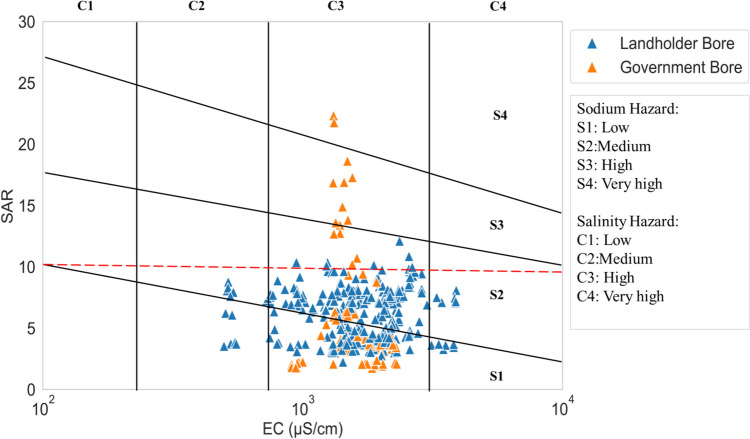
Wilcox diagram (SAR versus EC) of samples from landholder and government bores (2006–2023). A red dash line was added to highlight the SAR value of 10

When the KR of groundwater is less than one, it is deemed appropriate for irrigation; when the ratio is greater than one, it is not recommended for irrigation unless the local soils are suitable for an excess of sodium in the water (Greene et al. 2016). Most samples (93.4%) in this study had KR values > 1 and thus are not considered suitable for irrigation purposes. However, the Na, Ca, and Mg concentrations, which are used to calculate KR rations, comply with Australian standards for livestock (Table 1). This is the main agricultural use in the area and groundwater quality thus is suitable for livestock. Overall, landholder bores are within the range of acceptable quality for agricultural activities according to water quality assessment indices whereas monitoring bores screened in shallower sediments accounted for most of the unsuitable water sampled in this area (Figs. 3 and 4).

The SAR ratios provide insight into the extent to which other ions have replaced sodium, creating a sodium hazard. High sodium could influence soil permeability and lead to infiltration problems by Na^+^ replacing Ca^2+^ and Mg^2+^ on clay soils, and subsequent soil particle dispersion (e.g. Mohammed et al. (2022)). There are 20 groundwater samples (accounting for 4.7%) that had SAR values over 10, which are considered medium and high sodium hazards (Table 2). Of these samples, 14 samples were from government bores, which are not commonly used for agricultural activities. Overall, 95.3% of groundwater bores and 98.2% of landholder bores at OITC were in the low sodium hazard range (i.e. SAR < 10; Table 2). A Wilcox diagram (Wilcox 1955; Fig. 4) presents classifications of salinity hazard (EC) combined with SAR. Most landholder bores were in the C3-S1 and C3-S2 areas, in which sodium hazard is low to medium and salinity is in high range. Considering the natural salinity background of groundwater in the study area is 1000–3000 µS/cm (Bush 2009), a relatively high range is still acceptable. Overall, the groundwater at OITC can be considered suitable for irrigation purposes (Fig. 4 and Table 2). However, a small number of samples lies in the C3-S3 and C3-S4 sections indicating high sodium and high salinity, which indicate this water is not suitable for irrigation (Fig. 4). However, this is not a concern as these values were incidental from 2007 to 2015 datasets, and these samples were from government monitoring bores rather than landholder bores used for agricultural purposes.

### Recent geochemical trends at shallow monitoring piezometers

CO2CRC reference groundwater piezometers N1, O1, P1, and R1 were installed in 2019 for characterising near-surface geology and providing information to understand the impacts of fault on CO_2_ injection. The main aims of the drilling and installations were to obtain cored section of the Port Campbell Limestone, investigate characteristics of a shallow fault zone, establish the sequence’s geotechnical and hydrogeological properties, and construct groundwater monitoring wells and piezometers (Radke et al. 2022). Recent increases in Cl concentrations and TDS at CO2CRC shallow monitoring piezometers that were not observed at other monitoring sites (‘Long-term variations in groundwater quality’ section) warranted further monitoring.

**Fig. 5:**
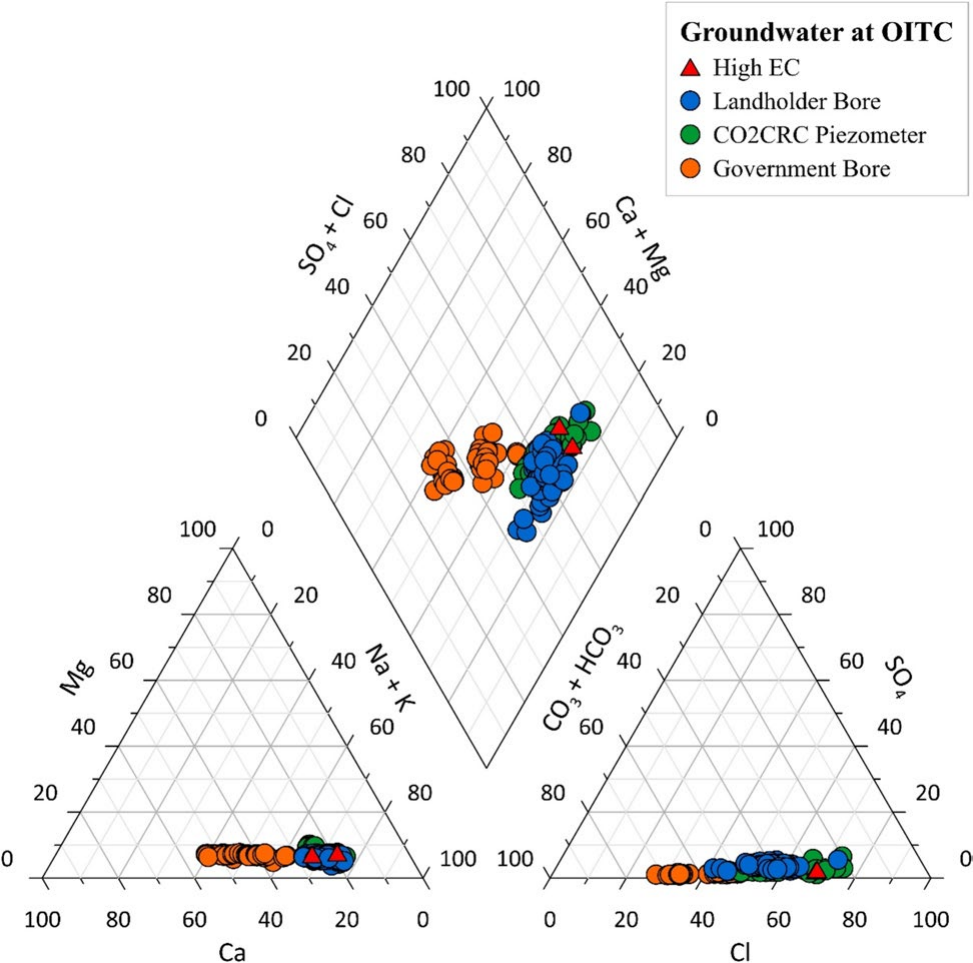
Piper diagram for selected groundwater samples (2006–2023). High EC values in 2023 in shallow monitoring piezometers were indicated by triangles

A Piper diagram shows the major ion composition of groundwater. High EC values measured at the piezometers in 2023 (O1 and R1 with values of 4118 and 5223 µS/cm respectively) are highlighted in Fig. 5. Groundwater from the government bores is dominantly Ca-Mg-HCO_3_ type. However, groundwater from piezometers showed a slight difference in type that Na-Cl-SO4 is the main type (Fig. 5). There is a transition visible trend between government bores and piezometers that Cl becomes poor to rich (Fig. 5). Na^+^ is the most abundant cation and Cl^−^ is the predominant anion in the CO2CRC piezometers. High Cl^−^ concentrations suggest evaporation or dissolution processes.

**Fig. 6:**
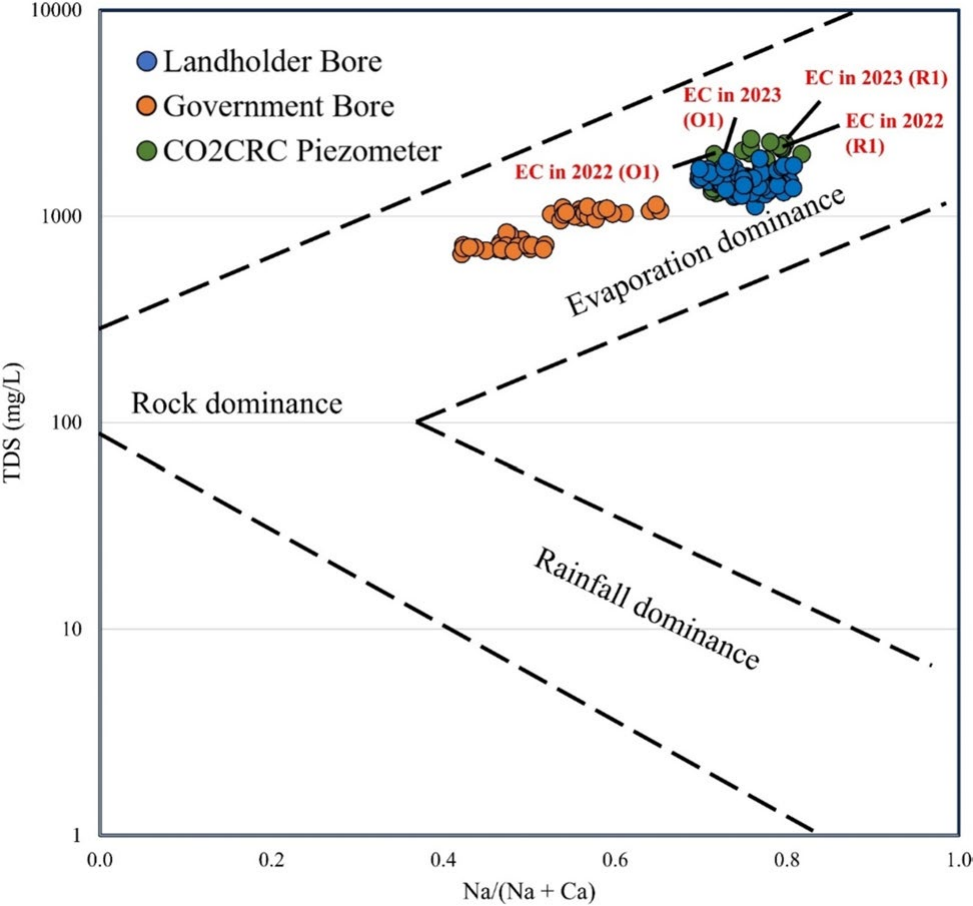
Gibbs plot of selected groundwater samples (2006–2023). High EC in 2023 (4118 and 5223 µS/cm) and their comparison with values in 2022 at shallow monitoring piezometers (O1 and R1) are highlighted

Gibbs plot was adopted to determine the major controlling factors of groundwater chemistry at OITC (Fig. 6). The plot demonstrated that the samples from CO2CRC piezometers and landholder bores plotted in the evaporation dominance zone, which is likely to be caused by dry climate conditions (e.g. Kumar et al. (2024)). The influence of evaporation or/and evapotranspiration is common in southeast Australia and consequently, the salinity of groundwater is increasing (e.g. Cartwright et al. (2019)). This is similar to implications from the piper plot above (Fig. 5). Some of the samples from government monitoring bores were in between the evaporation dominance and rock dominance zone (Fig. 6). This may indicate chemical rock weathering of minerals due to the dissolution of rock. Overall, the CO2CRC piezometers are more saline, and evaporation dominated compared to government monitoring bores. Especially for the high EC values in 2023 at piezometers O1 and R1 in the highlighted points slightly shift to the right side of plot when comparing with 2022 data at the same location (Fig. 6). This could indicate that the effects of evaporation or/and evapotranspiration may influence changes of salinity in piezometers due to their shallow (i.e. 1.72 m at O1 and 2.73 m at R1 in 2023) standing water levels (SWL). Other possible reasons for high salinity at these sampling locations include (1) downwards seepage of porewater that interacts with shallow and more saline sediments (Radke et al. 2022), or water affected by pasture land use; (2) salt near the ground surface mobilised downwards to groundwater by recharge.

### Naturally dissolved trace elements and water quality guidelines

Trace elements with analytical standards (increased to 58 elements since 2020) were generally at concentrations near or below the detection limit (LoD) in shallow groundwater, with several exceptions. Trace elements with maximum concentrations > 100 µg/L included Fe, Sr, Mn, and B. Trace elements with maximum concentrations < 100 µg/L and > 10 µg/L included Zn, Se, As, Li, and Ba. All other trace elements were at concentrations < 10 µg/L or below LoD including Pb and Cd (Zhou et al. 2023). It is noteworthy that Pb and Cd were at such low concentrations in shallow groundwater at this site, given potential concerns at other CCS sites where three elements (As, Se, and U) detected above a guideline value were discussed (Qafoku et al. 2017; Zheng et al. 2021), Australian and New Zealand Guidelines for Fresh and Marine Water Quality were used to compare with trace metal concentrations (ANZECC and ARMCANZ 2000; Table 3). The guidelines come with levels of species protection limits (95%) based on variable concentrations of specific elements. The selection of this limit is recommended for 95% species protection from toxicants in water in slightly to moderately disturbed systems (ANZECC and ARMCANZ 2000). This protection level means there is no significant change in water/sediment chemical and physical properties, including toxicants beyond natural variability.

**Table 3:** Summary of trace elements that have potential risks in the study area compared with Australian and New Zealand Guidelines for Fresh and Marine Water Quality

	As-III (µg/L)	As-V (µg/L)	Se (µg/L)	U (µg/L)
Min value	0.1		0.001	0.004
Max value	46.4		20.1	18
Average value	1.9		2.8	1.4
95% environmental protection value	24	13	11	n/a
LTV ^1^ for irrigation water	100		20	10
TV ^2^ for livestock water	500		20	200
Number of samples over 95% environmental protection	3	4	13	n/a
Percentage (%)	0.7	0.9	2.9	n/a
Number of samples over LTV	0	0	1	3
Percentage (%)	0	0	0.2	0.7
Number of samples over TV	0	0	1	0
Percentage (%)	0	0	0.2	0

^1^*LTV*, long-term trigger value

^2^*TV*, trigger value

Dissolved trace element results at this site between 2006 and 2022 were generally lower than guidelines for environmental protection and agricultural use, except for a few samples with naturally high selenium (Se) and arsenic (As) concentrations. Maximum As concentration of 46 µg/L from the groundwater sample exceeded the 95 protection guidelines for freshwaters either on As (III) or As (V). Only 3 and 4 samples (accounting for less than 1%) with concentrations above the protection level of As and these were from the piezometers. Se concentration was valued from 0.001 to 20.1 µg/L with an average of 2.83 µg/L and there are 2.9% of samples exceeding the 95% maximum limit (Table 2). While these samples were mainly landholder bores (61% from landholder bores and 39% from piezometers, respectively), most of the landholder bores had concentrations below 12 µg/L (one concentration unit over the recommended value), and only one bore was 20.1 µg/L.

It was found that U concentrations (0.004–18 µg/L) were very low, with 3 samples in 2007 and 2013 from one landholder bore exceeding guidelines for irrigation water. There is no relevant environmental guideline for U. However, since 2020, the concentration of U at this site decreased to below 3 µg/L. Overall, there is not currently an issue with dissolved trace metal concentrations because most of the sites exceeding the recommended values were recorded before 2020 since improved analytical techniques have enabled a more accurate detection limit.

### Dissolved trace element variation at the OITC and other CCS sites

#### Geochemical reactions of trace elements in geological strata

There is concern and speculation that mobilisation of trace elements could affect water quality in the near-surface environment at CCS sites with deep-well CO_2_ injection, which requires complex processed-based evaluation. A review of potential CO_2_ leakage impacts on groundwater quality parameters by Qafoku et al. (2017) noted that mobilisation of As, Pb, Ba, Se, and Cd may degrade groundwater quality, while a laboratory and modelling study by Bacon et al. (2016) observed trace metals release and mobilisation when interacting with the CO_2_-rich solution. However, there are no verified reports of degraded groundwater quality, such as due to trace element mobilisation, in shallow aquifers overlying CCS injection operations (‘Potential effects of CO2 on groundwater quality’ section).

At the OITC site, the few occurrences of high trace element concentrations in shallow groundwaters appear to be related to near-surface natural processes. Natural rainfall is slightly acidic (pH 5–5.6) and thus recharge of rainfall to the water table can result in geochemical reactions with geological strata. The trace element concentrations remain in the µg/L range with no multi-year upwards trend at any bore site (< 80 m depth), nor suggestion of geochemical processes that could be associated with CO_2_ injection operations to > 800 m depth. Trace element concentrations in the shallow sediment aquifer of the study area are likely very high (Radke et al. 2020), which is consistent with the fact that many minerals, including trace elements, bind to clay surfaces such as those that dominate the shallow lithology at the OITC. It is possible that old bores include pathways for groundwater ingress from the overlying unconsolidated sediments into the underlying limestone aquifer.

#### Likelihood for trace element migration from a deep injection zone

In contrast to the observations of trace elements in shallow aquifers due to natural processes at the OITC site, the likelihood of trace element migration from a deep injection zone involves distinct processes. A comprehensive trace metal content and potential for metal mobilisation during proposed CO_2_ injection into sandstone at > 2000 m depth in the Southern Surat Basin of Queensland was recently reported by Dawson et al. (2023). The study was based on reactive batch tests of rock core, rock digestions, and hydro-geochemical analysis and reaction path modelling and 3D reactive transport modelling including density flow of supercritical CO_2_. Trace metals including As, Pb, Cd, and others were mobilised at the low pH front of CO_2_-influenced water. However, As reactions followed a different path to the other trace metals, depending on O_2_ content of the migrating porewater. Trace metal concentrations reached hundreds of µg/L concentrations in the CO_2_-influenced water and decreased over time due to depletion in the source volumes, adsorption onto minerals, and advective mixing.

Importantly, Dawson et al. (2023) found that high concentrations of dissolved metals were not predicted beyond the CO_2_-influenced water that occurs near the depth of injection. CO_2_-influenced pore water and dissolved metals migrated hundreds of meters outwards laterally from the injection well over the 100-year simulations. Supercritical CO_2_ also moved vertically upwards along the dip of geological bedding and geological structures due to buoyancy effects, until low permeability strata prevented further migration towards the near surface. Dawson’s study highlighted that trace metal mobilisation requires comprehensive geochemical analysis and the results show the impact of trace metal values is most likely to be localised. However, at the OITC site, there is no evidence, in the long-term data available for this paper, that shallow groundwater is impacted by deep CO_2_ injection. The depth of CO_2_ interaction with groundwater below the zone of deep-well injection (> 800 m depth) and the associated reaction pathways and potential transport pathways thus require careful evaluation and modelling.

## Conclusion

Long-term shallow groundwater quality variations at a carbon capture and storage (CCS) site in Australia were evaluated for hydrochemical trends and in the context of groundwater quality assessment indicators/guidelines for agricultural usage. There was robust evidence for stable groundwater quality in shallow aquifers over 15 years of OITC operations, despite natural background variability. Some of the temporary changes observed in salinity, pH, or dissolved trace metals were at monitoring groundwater bores/piezometers in the near surface, with groundwater that is not used for agriculture. The detection of As, Se, and U at a few sites was considered in the context of water quality guidelines for the potential mobilisation of dissolved trace metals. It was demonstrated that these water quality changes were a result of natural processes and did not pose risks to the local environment.

Importantly, the shallow groundwaters at OITC remained generally suitable for livestock and irrigation, benefiting agricultural enterprises in the area overlying and near the site. Water quality indices at OITC (e.g. SAR, KR index) were generally suitable for irrigation depending on soil interactions with Na and K. Approximately 58 trace elements were generally at concentrations near or below new lower detection limits (LoD) (since 2020) and complied with water quality guidelines for agriculture, with a few exceptions.

These long-term results demonstrated that shallow freshwater aquifers are not affected by deep-well injection into deep confined formations at this site. There is no evidence of changes in shallow groundwater quality for stock and irrigation use that could be traced to CO_2_ injection to storages at > 800 m depth reported at any operational CCS site around the world. Again, it was beyond the scope of this paper to present a comprehensive hydrogeochemical evaluation (e.g. including tracers) of the unlikely possibility of migration of CO_2_ upwards from the target reservoir. For the first time, this paper has presented long-term monitoring and assurance of near-surface environments at CCS sites in the context of stakeholders who rely on shallow groundwater for agriculture.

## Data Availability

Datasets generated during the current study are available from the corresponding author on reasonable request.
